# Fluoride contributes to the shaping of microbial community in high fluoride groundwater in Qiji County, Yuncheng City, China

**DOI:** 10.1038/s41598-019-50914-6

**Published:** 2019-10-09

**Authors:** Xin Zhang, Xubo Gao, Chengcheng Li, Xuesong Luo, Yanxin Wang

**Affiliations:** 1 School of Environmental Studies & State Key Laboratory of Biogeology and Environmental Geology, China University of Geosciences, Wuhan, 430074 P.R. China; 2 College of Resources and Environment, Huazhong Agricultural University, Wuhan, 430070 China

**Keywords:** Water microbiology, Element cycles

## Abstract

As a toxic element, excessive amounts of fluoride in environment can be harmful because of its antimicrobial activity, however little is known about the relationship between fluoride and the bacterial community in groundwater systems. Here, we use samples from a typical fluorosis area to test the hypothesis that fluoride concentration is a fundamental structuring factor for bacterial communities in groundwater. Thirteen groundwater samples were collected; high-throughput 16S rRNA gene sequencing and statistical analysis were conducted to compare the bacterial community composition in individual wells. The results showed that *Proteobacteria*, with most relative abundance in groundwater, decreased along the groundwater fluoride concentration. Additionally, relative abundances of 12 families were also statistically correlated with fluoride concentration. The bacterial community was significantly explained by TOC (P = 0.045) and fluoride concentration (P = 0.007) of groundwater. This suggests that fluoride and TOC likely plays an important role in shaping the microbial community structure in these groundwater systems. Our research suggest that fluoride concentration should be taken into consideration in future when evaluating microbial response to environmental conditions in groundwater system, especially for fluoride rich groundwater.

## Introduction

Fluoride is a toxic element that can cause fluorosis to organisms^1,2^ whether excessive or deficient. Recently, it has also been identified as a fundamental factor impacting microbial activity and communities in environment^3,4^ due to its potential antimicrobial activity^5,6^. One piece of evidence for the antimicrobial activity is the anticaries actions of fluoride by affecting bacterial metabolism^7^. It was found that ureolysis by urease-positive bacteria (*Staphylococcus epidermidis*, *Streptococcus salivarius* and *Actinomyces naeslundii*) was inhibited by fluoride in suspensions or mono-organism biofilms at plaque levels of 0.1–0.5 mm in a pH-dependent manner^8^. Fluoride, in the form of a complex such as SnF_2_, has shown a significant effect against oral micro-organisms *in vivo* and *in vitro*^9–11^.

Additionally, numerous studies have reported on the ability of fluoride, normally as sodium fluoride, to cause changes to microbial activity and community composition in soil^4,12^. Fluoride-induced changes of chemical properties and microbial activities in humus soils were reported by Wilke^13^; it was shown that dehydrogenase, alkaline phosphatase and arylsulfatase activities as well as nitrification were inhibited at much lower F-additions in the moder and mor soils. The inhibition of fluoride on the activity of soil peroxidase and ATPase was further confirmed by Reddy and Kaur^14^ and Yadu *et al*.^15^. The negative impact of fluoride on soil microorganisms was quantified by Rao and Pal^16^, who reported that elevated fluoride concentrations (380–1803 mg/g soil) were found to inhibit microbial growth and activity and decomposition of organic matter. Lately, inhibition of soil respiration and dehydrogenase activity was observed when fluoride concentrations in soils were below 200 mg/g, whereas fluoride concentrations of 200–2000 mg/g inhibit denitrification in soils^17^. Currently, the information about minimum fluoride concentrations in soils, affecting the activity of soil microorganism, is inconclusive. However, the presence of fluoride, especially in amounts significantly exceeding its natural concentrations in soil, inhibits microbial growth and the decomposition of organic matter^18–20^.

Based on the previous studies, the antimicrobial activity of fluoride is mediated via three major effects of fluoride: (i) enzyme inhibition. The direct inhibition of the F-ATPase enzyme by fluoride was found to be dependent on cations^21,22^, and fluoride is a potent inhibitor of enolase, catalase, phosphatases and other enzymes of many organisms^23–25^; (ii) fluoride was found to alter the proton movement through cell membranes and inhibit intracellular acid production^26,27^; (iii) fluoride can inhibit certain microbes in the ingestion, transformation and utilization of certain nutrients, affecting the synthesis of extracellular polysaccharides and the storage of intracellular polysaccharides^28–30^.

As a toxic element, fluoride influences the metabolic behavior of microbes in natural environment. According to Mendes *et al*., during the dissolution of rock phosphate by *Aspergillus niger*, fluoride was capable of decreasing fungal growth, citric acid production, and acidification^31^. In an indoor culture, Bradshaw *et al*. found that a continuous supply of 1 mM NaF reduced the viable counts of microflora in solution at pH 7^32^. It was reported that the per liter concentration of fluoride ranges from several micrograms to dozens of milligrams to even one thousand milligrams in groundwater^33–36^. While groundwater systems (groundwater-sediment) contain large numbers of microorganisms^37,38^. However, how microbial communities in groundwater respond to different fluoride levels is not yet well understood. We hypothesize that fluoride influences microbial community diversity and structure by its toxic effects on microbes with various [F] in groundwater.

A typical area with endemic fluorosis, Qiji County of Yuncheng City, China^39–42^, was chosen as a model system in which to test our hypothesis. According to Li *et al*., the fluoride concentration ranges from approximately 1.4 mg/L to 14.2 mg/L in Qiji County^41^, exceeding the World Health Organization (WHO) limit of 1.5 mg/L in drinking water^43^. And over 50% were meeting or exceeding the levels of fluoride that were reported to be needed to kill bacteria, approximating 0.16 to 0.30 mmol/L (3.04 to 5.70 mg/L)^44^. To obtain samples with various fluoride concentrations, shallow groundwater samples were collected from thirteen different wells for a coupled study of both fluoride and microbial community. The aim of this study is (i) to investigate the microbial community in groundwater of the area; (ii) to discern which environmental parameters significantly influence the microbial community at the phylum, family and OTU levels; and (iii) to discuss the potential effects of F/[F] on the groundwater microbial community.

## Materials and Methods

### Site description

Qiji County is located in the southwestern part of Yuncheng City, Shanxi Province, China^45^ and covers an area of 77 km^2^ (between 34°40′ and 35°02′N and 110°29′ and 110°30′E). The region has a semi-arid climate with most of the rainfall (>65%) occurring between June and October. The Quaternary sediment is composed of primarily aeolian loess, lacustrine clays, fluvial sands and gravels in the area. The major shallow aquifer consists of an admixture of fine, to medium grained and coarse sands with depth between 35 to 65 meters in the area. The regional shallow groundwater flows from the northwest to the southeast, toward the salt lake^39,41^. Historically, approximately 50% of the people living in the area have been affected by fluorosis in the past several decades. Long-term intake of high-fluoride ground water is the major reason for endemic fluorosis in Yuncheng Basin^39,46,47^ and other sites in the world^2^. The Yuncheng basin has a semi-arid climate with a strong evaporation. Groundwater is the major source of water drinking causing the lack of surface water. The fluoride rich groundwater normally located in the low land areas in the basin. The chemistry of the groundwater with high fluoride concentration is characterized as: Na-rich and Ca-poor, with high pH and HCO_3_^−^ values and low TDS values^39,41,45,48^. The dissolution of fluorine-bearing minerals, cation exchange and evaporation were main factors that control the occurrence of high fluoride shallow groundwater.

### Sample collection and geochemical analysis

A total of thirteen water samples were collected in August, 2014, from the power - operated wells around the borehole located in Qiji area, Yuncheng City, Shanxi Province (Table 1). When sampling, water samples were collected only after the *in situ* physicochemical parameter, including pH, redox potential (Eh), dissolved O_2_ (DO), temperature (T), and electrical conductivity (EC), were stable. These four parameters had been measured using portable HACH EC, DO and pH meters. Subsamples for chemical analysis were filtered through 0.45-μm filters and collected in three acid-washed, high-density polyethylene (HDPE) bottles that had been rinsed with the sample water thoroughly (at least three times before sampling). Alkalinity measurements were performed by pH-verified colorimetric titration. The microbial samples were collected by filtration of 5–10 L of water through 0.22-μm filters, and the biomass-containing filters were wrapped and immediately stored at −80 °C for the analysis of biodiversity. The other hydrogeochemical analyses were performed at the State Key Laboratory of Biological and Environmental Geology, China University of Geosciences (Wuhan). The concentrations of Cl^−^, SO_4_^2−^, NO_3_^−^, and F^−^ were determined using ion chromatography (IC) (Dionex 120, Dionex, Sunnyvale, CA, USA). Samples for cation analysis were acidified with trace metal grade HNO_3_ to pH less than 2.0. Major cations (K^+^, Na^+^, Ca^2+^ and Mg^2+^) were measured using inductively coupled plasma-atomic emission spectroscopy (ICP-AES) (IRIS Intrepid II XSP, Thermo Elemental, Madison, WI, USA). The instrumental detection limit was 0.06 ppm. The standards used for the ICP-AES calibration were within 5% of external standards. Samples for total organic carbon (TOC), total nitrogen (TN) and total phosphorus (TP) analysis were acidified with H_2_SO_4_. Sub-samples for TOC were analyzed with a TOC analyzer (Analytik Jena, Multi N/C, 3100); samples for TN and TP were analyzed with ultraviolet spectrophotometer (HITACHI U-1900).

**Table 1:** Geochemical parameters of groundwater samples (n = 13) with variable fluoride concentration in Yuncheng Basin.

Sample	Longitude	Latitude	pH	Well Depth	EC	HCO_3_^−^	NO_3_^−^	Cl^−^	Ca^2+^	Mg^2+^	SO_4_^2-^	Na^+^	K^+^	F^−^	TOC	TN	TP	DO
				m	μs/cm	mg/L	mg/L	mg/L	mg/L	mg/L	mg/L	mg/L	mg/L	mg/L	mg/L	mg/L	mg/L	mg/L
QJ01	35.0008°	110.5629°	8.39	40	1029	583.4	37.38	58.49	4.49	8.94	47.23	269.2	0.40	5.23	0.12	37.38	0.05	6.46
QJ02	35.0235°	110.5800°	8.50	60	1154	615.1	18.87	56.72	2.73	9.04	86.02	310.6	0.31	8.07	0.84	18.87	0.24	6.37
QJ03	35.0202°	110.5114°	8.16	65	908	573.6	8.46	30.84	1.92	5.54	51.46	248.0	0.32	6.66	1.18	8.46	0.14	8.64
QJ04	35.0207°	110.5328°	8.43	40	879	574.8	8.28	13.12	1.76	6.22	27.26	244.5	0.24	8.47	1.28	8.28	0.18	6.91
QJ05	34.9962°	110.5313°	8.39	50	1046	673.7	11.99	31.90	8.18	4.96	34.18	282.2	0.32	6.05	2.33	11.99	0.16	4.78
QJ06	35.0188°	110.5087°	8.46	40	885	548.0	28.43	23.75	2.08	5.64	31.1	245.7	0.25	8.89	1.46	28.43	0.16	7.20
QJ07	34.9820°	110.5233°	8.09	40	1170	561.4	58.57	102.8	28.86	19.44	136.3	273.5	0.38	3.05	1.36	58.57	0.06	6.85
QJ08	34.9824°	110.5080°	8.31	50	1209	661.5	7.81	73.74	4.01	10.21	59.14	310.6	0.36	4.45	2.49	7.81	0.07	7.50
QJ09	34.9742°	110.4870°	8.15	40	1888	668.8	53.33	184.3	12.02	37.67	211.2	387.5	0.48	5.12	2.73	53.33	0.18	5.83
QJ10	34.9783°	110.5361°	7.78	45	3280	951.9	16.75	452.0	30.06	82.64	697.0	794.7	0.64	3.51	4.30	16.75	0.02	6.03
QJ11	34.9768°	110.5742°	7.69	35	4440	1128	66.36	651.4	34.07	138.3	757.4	908.1	3.63	4.45	3.19	66.36	0.04	5.49
QJ12	34.9772°	110.5800°	8.00	35	3190	793.3	17.53	505.2	20.04	68.06	685.4	803.0	0.62	5.9	4.38	17.53	0.02	6.76
QJ13	34.9811°	110.5903°	7.81	35	3510	1031	23.73	487.4	32.06	100.9	616.3	771.8	1.29	4.88	3.69	23.73	0.02	7.78

db, below detection limit.

### DNA extraction, PCR amplification, and sequence analysis

Total genomic DNA was extracted from the filtered groundwater samples (0.22 μm, millipore) at the sampling site. DNA extractions were conducted using the PowerWater®DNA Isolation kit (Anbiosci Tech LTD, 14900–50-NF) according to the manufacturer’s manual. DNA yield was quantified with Nanodrop (Thermo Scientific NanoDrop 2000) and then stored at −80 °C until PCR amplification. PCR amplifications were performed with the 515f/806r primer sets (The reverse primer contains a 6-bp error-correcting barcode to distinguish sequences that originated from different samples) that amplifies the V4 region of the 16S rDNA gene, following the protocol described previously^49^. Sequencing was conducted with 200 ng of amplified product on an Illumina MiSeq platform at Novogene (Beijing, China).

Sequence analysis was performed with the UPARSE software package using the UPARSE-OTU and UPARSE-OTUref algorithms^50^. Sequences were further denoised using conduct filter analysis by applying the microbial community analysis software QIIME^51^. High-quality sequences were obtained and compared with the data library (Gold database, http://drive5.com/uchime/uchime_download.html), and the chimera sequences were measured and removed^52^ (UCHIME Algorithm, http://www.drive5.com/usearch/manual/uchime_algo.html). This output was clustered using Uparse^53^ software at the 97% sequence identity level, resulting in 6383 operational taxonomic units (OTUs). A representative sequence from each OTU was classified using the Ribosomal Database Project (RDP) classifier^54^ (Version 2.2, http://sourceforge.net/projects/rdp-classifier/) and GreenGene data set^55^ http://greengenes.lbl.gov/cgi-bin/nph-index.cgi). The OTUs table was formatted to allow comparisons using β diversity metrics.

### Statistical analysis

Statistical analyses were performed in QIIME and using package “vegan” in R, version 3.2.4 (https://www.r-project.org/)^56,57^. Rarefaction curves, species richness estimators and community diversity indices were calculated in QIIME. The Chao1 richness index, Shannon diversity index and coverage rate were calculated to reflect the alpha-diversity of samples^41^.

A heat map of abundance data at the class level was constructed. Two kinds of analyses were performed to investigate the relationships between the microbial community (at the OTUs level) and the geochemical parameters: (1) Mantel tests were used to examine the relationship between the bacterial community structure and the geochemical parameters^58^. The significance of each environmental factor was tested by analysis of variance with 999 permutations. Mantel tests were performed with both weighted Uni Frac and Bray Curtis distance matrices for the microbial community data and distance matrices (Euclidian distance) for each environment factor. (2) Redundancy analysis (RDA) with forward selection to identify the factors that could best explain the variation in the microbial community^56^ and significance was tested by a Monte Carlo permutation test based on 999 random permutations. Some additional correlation analyses were performed using SPASS software.

## Results

### Groundwater geochemistry

The groundwater was slightly alkaline (7.69–8.50) and fresh to slightly saline (Table 1). Fluoride concentration in groundwater varied from 3.05 to 8.89 mg/L, which was within the range reported by others^41^ from the area. The TOC values ranged from 0.12 mg/L to 4.38 mg/L. Na^+^ was the most dominant cation, with concentrations of 244.5~908.1 mg/L, followed by Mg^2+^, Ca^2+^ and K^+^. Bicarbonate and sulfate were major anions, with concentrations of 548.0~1128 mg/L and 27.26~757.4 mg/L, respectively. This results were resistant with that Na-rich, Ca-poor and high bicarbonate concentration were the mainly characteristic of groundwater in the basin^41,45^. The correlation analysis (Supplementary Table 1) showed that the fluoride is significantly correlated with pH (R = 0.678, P < 0.05), TP (R = 0.730, P < 0.01) and Ca^2+^ (R = −0.720, P < 0.01). Under high pH alkaline conditions, OH^-^ could exchange with F^−^ adsorbed on minerals and soil^59,60^ and prevent F^−^ from complexing with cations, which results in a high release of F^−^ in groundwater. The positive correlation between fluoride and TP may partly due to the contamination of the phosphatic fertilizer and the competitive adsorption by PO_4_^3−^ which could cause the desorption of fluoride from mineral/organic matter surfaces within the groundwater system^61^. Meanwhile, the negative correlation between [F] and [Ca] is attributed to the dissolution/precipitation equilibrium of major fluoride bearing minerals, especially fluorite (CaF_2_)^45^. It is suggested that the concentration of fluoride in groundwater is under the control of natural water-sediments rock interactions (e.g., cation exchange, competitive adsorption, dissolution/precipitation) in natural groundwater systems^62^.

### Alpha diversity of bacterial community

**Figure 1:**
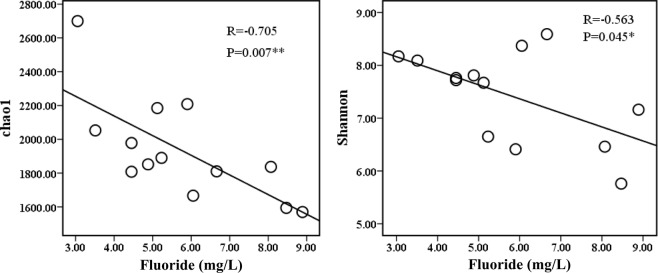
The Chao1 and Shannon index along a fluoride concentration gradient.

A total of 329889 valid reads (average read length, 253 bp) were identified from thirteen Illumina sequencing libraries after filtering out the low-quality reads and chimeras and trimming the adapters, as well as bar codes and primers. The values of the Chao1 index ranged from 1569 to 2699 while the values of Shannon diversity index ranged from 5.76 to 8.59 (Supplementary Table 2). Obvious change in groundwater community diversity was apparent across the fluoride concentration gradient (Fig. 1). The microorganism communities in samples with high fluoride concentration were substantially simpler than those in samples with medium fluoride concentration. Besides, [Ca], [NO_3_] and [TN] had significant positive relationship with Chao 1 (Supplementary Table 3). Significant negative correlations were observed between fluoride concentration with Chao1 (R = −0.705, P = 0.007, Fig. 1) and Shannon (R = −0.563, P = 0.010, Fig. 1), which may be interpreted by the inhibitive ability of fluoride toward micro-organisms in groundwater in the area.

### Bacterial community composition

**Figure 2:**
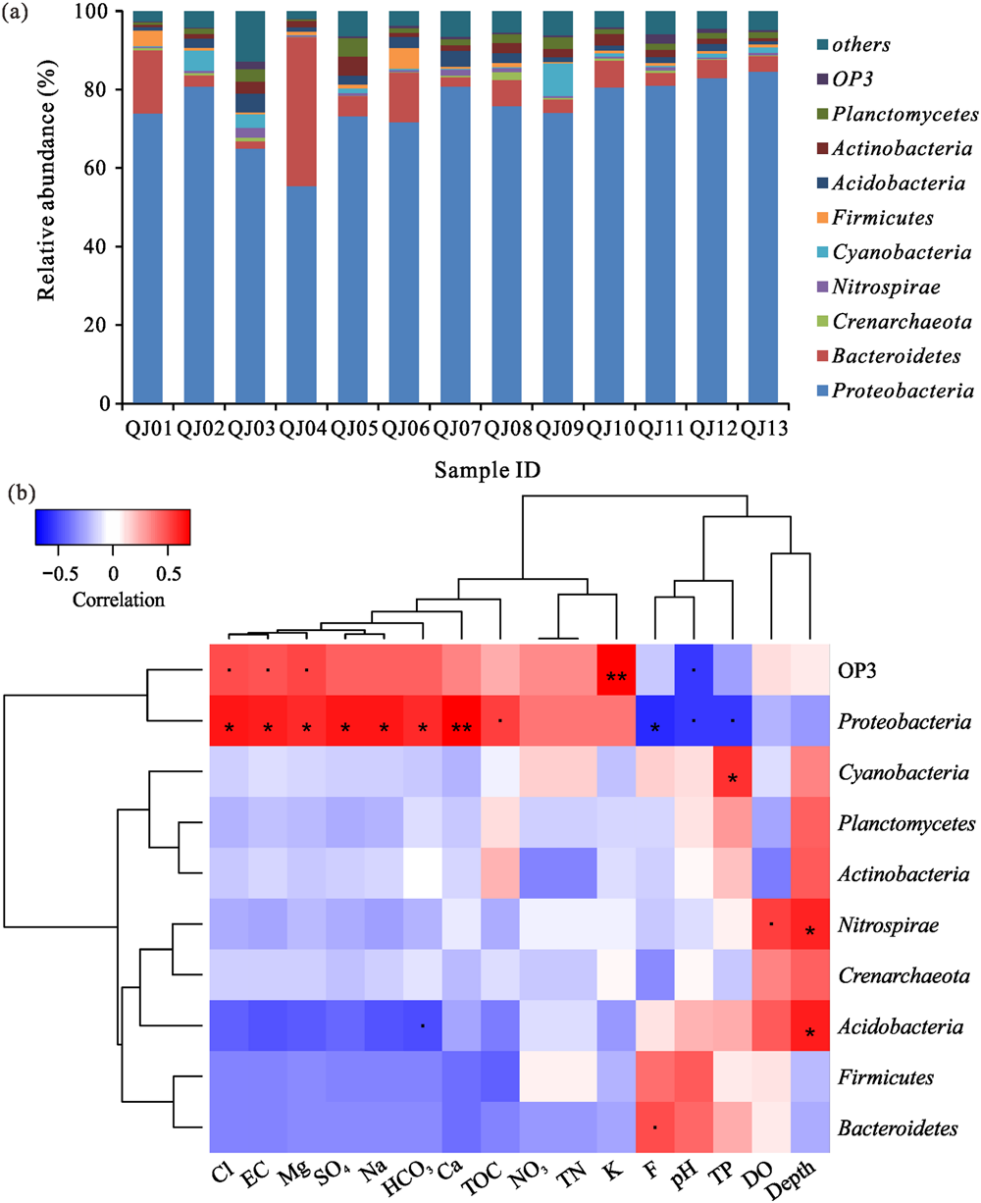
Microbial community composition at phylum level in the study area. (**a**) Taxonomic classification of bacterial reads retrieved from different groundwater samples at the phylum level using the RDP classifier. “others” indicates phyla with relative abundances of less than 1.0%; (**b**) Heat map of correlation between the ten most abundant bacterial phyla and hydrochemical factors. Significance codes: 0 < p < 0.001 “***”, 0.001 < p < 0.01 “**”, 0.01 < p < 0.05 “*”, 0.05 < p < 0.1 “•”.

The most abundant prokaryotic organisms in groundwater samples belonged phylogenetically to the *Proteobacteria* phylum, accounting for 55.41% (in QJ04) to 84.58% (in QJ13) of the total valid reads in all libraries (Fig. 2a, Supplementary Fig. 1). The second most abundant prokaryotic organisms were *Bacteroidetes* (1.86% to 37.95% of the total valid reads), followed by *Cyanobacteria* (0.11% to 8.32%). *Crenarchaeota*, *Nitrospirae*, *Firmicutes*, *Acidobacteria*, *Actinobacteria*. *Planctomycetes* and OP3 were found to be the minor phyla. The microbial community, at the phylum level, was similar among groundwater samples (QJ07, QJ10, QJ08, QJ11 and QJ13) with a medium fluoride concentration (3.05–4.88 mg/L). Significant differences in bacterial community were observed among the rest of samples with a higher fluoride concentration (5.12–8.89 mg/L). Frequently, fluoride-related changes in the relative abundances of bacterial phyla were observed. For example, *Proteobacteria* occurred at lower abundances in the libraries from groundwater samples with higher fluoride concentration than that from samples with a medium F concentration (Supplementary Fig. 2). While the abundance of *Proteobacteria* was positively related with EC, TOC and major ions (Fig. 2b).

**Figure 3:**
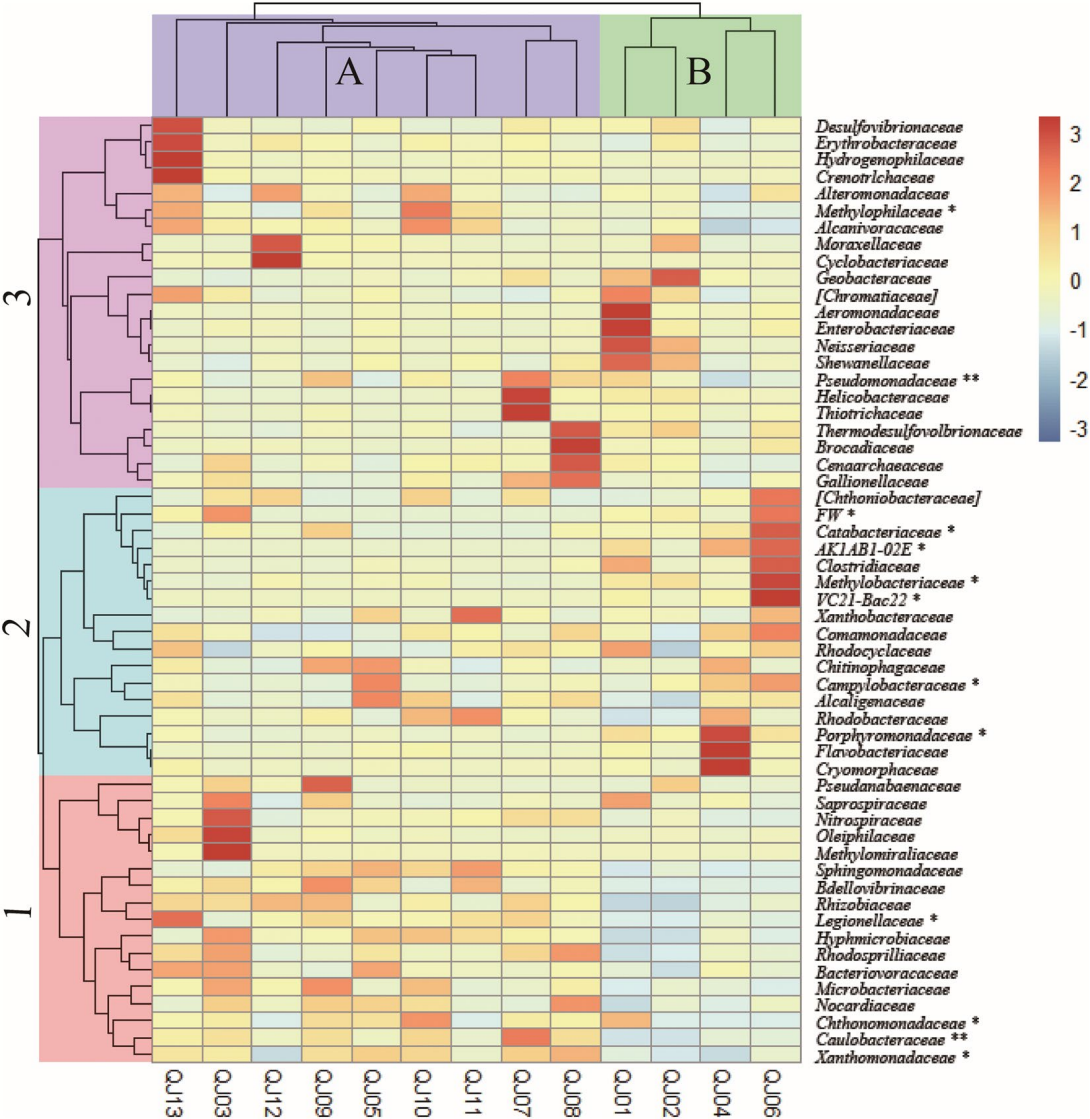
Heat map of log relative abundances of the distribution of dominant family in the three samples. The double hierarchical dendrogram shows the microbial distribution of the 13 samples. The log relative abundance for the microbial families are depicted by the color intensity; the color key is at the topright. The microbial families with * at their right are related with fluoride concentration analyzed through SPASS software, denoted **(p < 0.01) and *(p < 0.05).

The correlation analysis conducted with heat maps involved a clustering analysis based on the distribution of the predominant families, and deeper colors represent higher richness (Fig. 3). The column cluster illustrated that most of the samples with higher fluoride concentrations (QJ02, QJ04, QJ06) and one sample with medium fluoride concentration (QJ01) were clustered together (Cluster B). Bacterial communities under lower fluoride concentration (QJ07, QJ08, QJ10, QJ11, QJ05, QJ09, QJ12 and QJ13) conditions were clustered into another group (Cluster A). The dominant family was grouped into three groups. Samples in the Cluster B, with a higher fluoride concentration, have a lower relative abundance of families (soft color) in group one and part of the families in group three (from *Desulfovibrionaceae* to *Alcanivoracaceae* at the top of the heat map). In contrast, the samples in Cluster A, with a medium fluoride concentration, have a higher relative abundance of families (deep color) in group one and part in group three. Lower relative abundances of the families *Xanthomonadaceae*, *Caulobacteraceae*, *Chthonomonadaceae*, *Nocardiaceae*, *Microbacteriaceae*, *Bacteriovoracaceae*, *Rhodasprilliaceae*, *Hyphmicrobiaceae*, *Legionellaceae*, *Rhizobiaceae*, *Bdellovibrinaceae* and *Sphingomonadaceae* were observed in the high fluoride groundwater, while higher relative abundances of these families were observed in groundwater with medium fluoride concentrations. Obviously, the richness of the predominant families shows a significant diversity in groundwater with different fluoride concentration levels.

**Figure 4:**
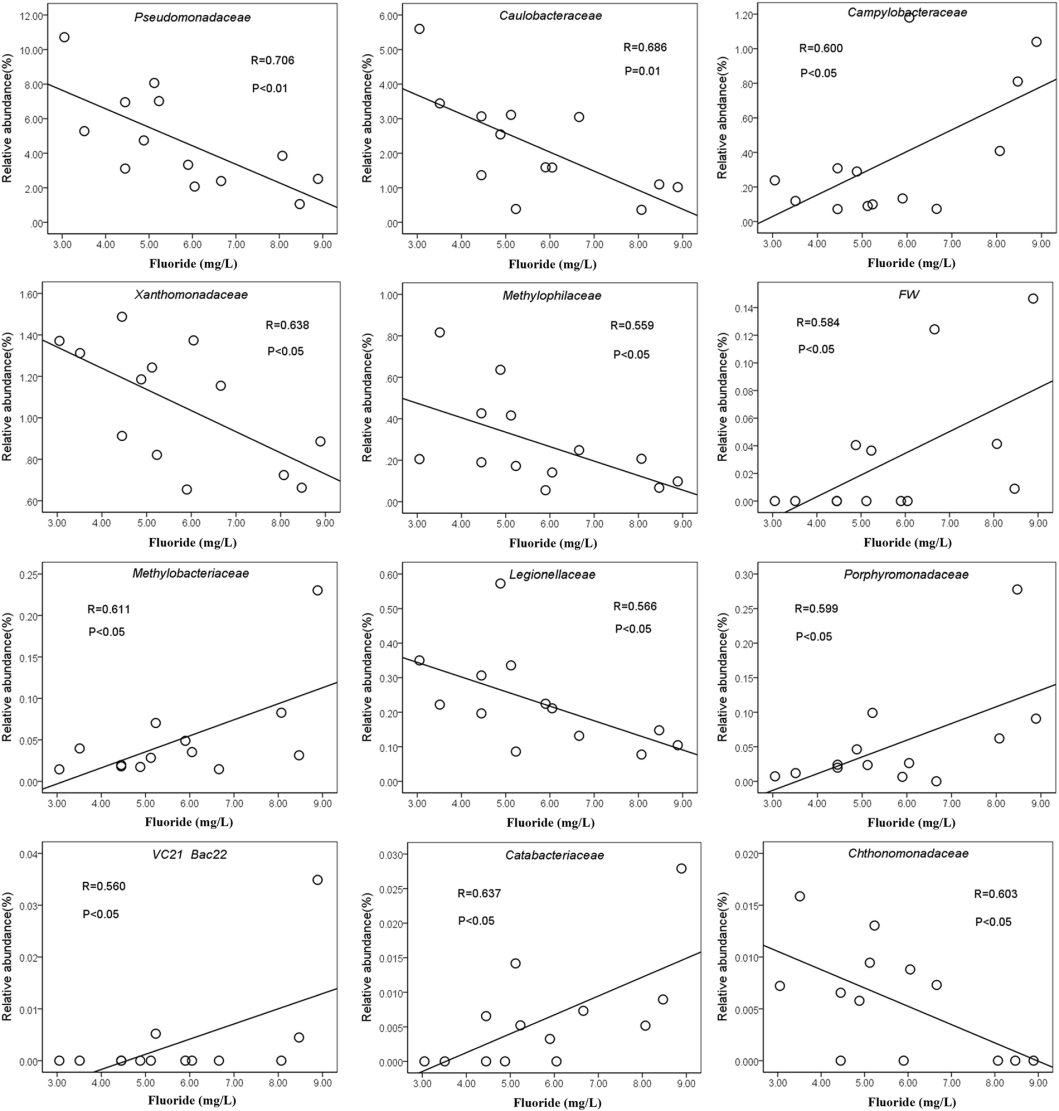
The relative abundance of microbial family along fluoride gradients. Correlation analyses and significance test for species at family level were conducted using SPASS software.

Figure 4 shows the relative abundances of bacterial families across the fluoride concentration. The relative abundances of *Pseudomonadaceae* and *Caulobacteraceae* were negatively correlated with fluoride concentration at significant level (p < 0.01). The relative abundances of *Xanthomonadaceae*, *Methylophilaceae*, *Legionellaceae* and *Chthonomonadaceae* were also negatively correlated (p < 0.05) with fluoride concentration. Additionally, the relative abundances of these families, excepting *Methylophilaceae* and *Legionellaceae*, did not show any significant correlations with any other parameters (Supplementary Table 4). Therefore, it is cautiously inferred that the decrease of the relative abundance of these families may be attributed to the increase of fluoride concentration in groundwater. It was also shown that the relative abundances of *Campylobacteraceae*, *Methylobacteriaceae*, *Porphyromonadaceae*, *Catabacteriaceae* and two uncultured lineages designated FW and VC21-Bac22 were positively correlated (p < 0.05) with fluoride concentration. This implied that some of the families are resistant to a certain higher concentration of fluoride in groundwater.

### Relationships of bacterial community and hydrogeochemical parameters

**Figure 5:**
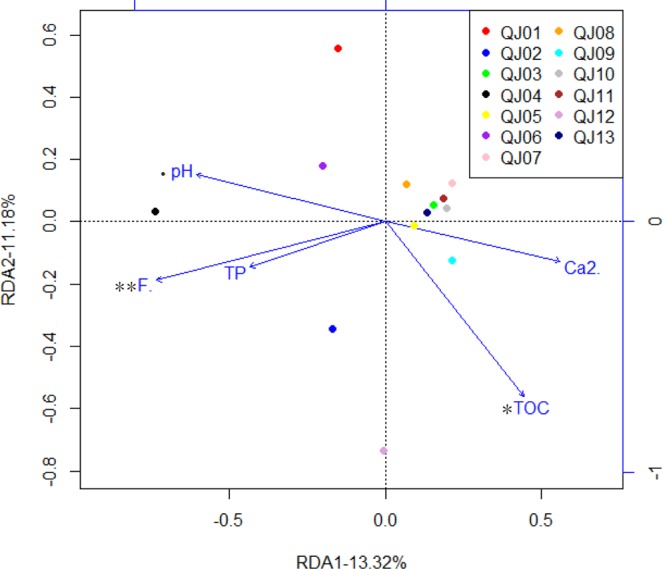
RDA biplot of bacterial community composition and the five most significant geochemical parameters. Each dot represents one sample. Arrows indicate the direction and magnitude of geochemical parameters associated with bacterial community structures. Total variance is 0.027, and eigenvalues for constrained axes, RDA1 and RDA2, are 0.0036 and 0.0030, respectively. The significant vectors in the figure were highlighted to avoid misunderstanding. Significance codes: 0 < p < 0.001 “***”, 0.001 < p < 0.01 “**”, 0.01 < p < 0.05 “*”, 0.05 < p < 0.1 “•”.

The differences in microbial community structure could be influenced by hydrogeochemical parameters. For RDA analysis, we employed forward selection by performing a Mantel test with the number of permutations being 999. Then, we drew the bacterial diversity diagram at OTU level with five selected geochemical parameters (pH, Ca^2+^, F^−^, TOC and TP) that best explained the variance of the bacterial communities in the thirteen groundwater samples (Fig. 5). The variance inflation factors of these five parameters were all less than 20.

The first axis of the RDA explained 13.32% of the variance of bacterial community and was positively correlated with the samples at a medium fluoride concentration except QJ12 and negatively correlated with the samples with a higher fluoride concentration. Interestingly, the samples (except QJ12) with a medium fluoride concentration grouped together in the RDA profile. These five hydrogeochemical parameters explained 36.6% of the total variance. TOC and fluoride concentration explained the variability of bacterial community at the significance level (r^2^ = 0.45, p = 0.045 vs. r^2^ = 0.59, p = 0.007, Supplementary Table 5). While the effects from pH could be present but only at the marginal significance level (r^2^ = 0.40, p = 0.067). Ca^2+^ and TP were insignificant factors.

## Discussion

Knowledge of microbial communities in relation to hydrogeochemical and environmental conditions is important for understanding biogeochemical processes and the mobilization of pollutants (solutes) in groundwater systems^63^. Microbial community diversity and structure are affected by many environmental factors, such as pH, and the concentrations of various elements, electron donors and acceptors in soils and groundwater systems^64,65^. It has been reported that fluoride led to a decrease in microbial biomass in a water treatment system^20^ and inhibited the activity of soil dehydrogenase, arylsulfatase, alkaline phosphatase, acid phosphatase, peroxidase and ATPase^13–15^; it is likely that fluoride had a negative impact on the function of the microbial community. Therefore, we speculated that F may alter the microbial community in groundwater. In this case study, the Chao1 index and Shannon diversity were significantly and negatively correlated with fluoride concentration in the study wells. This indicated that fluoride may have a strong effect on the diversity of bacterial communities in this study.

As a toxic element, fluoride can inhibit or kill microbes by affecting bacterial metabolism through a set of actions with fundamentally different mechanisms^21,26,27,29^. For example, Al-F or Mg-F, the major metal-fluoride complexes present in high fluoride groundwater, were responsible for fluoride inhibition of phosphate transfer enzymes under laboratory conditions^66^. This suggests that fluoride, in the form of metal-fluoride complexes, is a possible enzyme inhibitor in groundwater. Previous studies have shown that most of the fluoride movement across the cell membranes of bacteria would be of the HF form, even at pH 7^7^. In groundwater, an increase of fluoride concentration possibly cause more fluoride to move across the cell membrane in the form of HF. Accumulation of fluoride in bacteria is largely dependent on its binding to various cell structures, mainly proteins. In the cytoplasm, the dissociation of HF yields F^−^ and H^+^, which can act as a metabolic inhibitor and reduce the acid tolerance of the bacteria^67^. Indeed, our data were also in agreement with this mechanism of F action as the relative abundance of *Proteobacteria* decrease in the high fluoride sample, suggesting the presence of a toxic effect from F.

Bulk geochemical parameters, such as pH, fluoride, major ions, TOC and TP have been shown to explain variance in microbial communities in groundwater. The variance in the microbial community in our study, however, was best explained by TOC and fluoride. Numerous studies have shown that microbial community can be strongly correlated with TOC^52,68,69^. Our study and others are in agreement with the fact that carbon, either organic carbon or inorganic carbon, is a necessary nutrient element for microorganisms and is one of the fundamental factors that structures microbial communities by controlling the growth and distribution of microorganism.

The RDA also indicated that fluoride was the most significant factor influencing the bacterial community in groundwater at the OTU level. In this case study, distinct bacterial communities could be formed between individual wells due to the differences of fluoride concentrations. First, frequent fluoride-related changes in the relative abundance of *Proteobacteria* were observed in the study wells. In addition, at the family level, fluoride concentration was significantly positively or negatively correlated with the relative abundances of twelve families in groundwater. It was reported that some prokaryotes (bacteria and actinomycetes) were resistant to the high concentrations of F compounds^70^. Genus *Pseudomonas*, *Bacillus* sp., *Acinetobacter* sp. and *Streptococcus* sp. were reported to be resistant to fluoride by an ancient system composed by fluoride-specific riboswitches and commonly associated proteins such as CrcB^71–74^. Besides, *Methylobacterium extorquens* DM4, encoding at least 10 fluoride riboswitches in its genome, might consume fluorinated hydrocarbons a food source with a robust fluoride sensor and toxicity mitigation response system^71^. This may partly explain why some families were in positive correlation with fluoride concentration. In groundwater ecosystem, microbes are motile (by itself or flow) and faced dynamic biogeochemical conditions which were possibly challengeable^75^. Stochastic processes frequently influences the assembly of microbial communities, while the selective pressures might drive changes in microbial community composition via deterministic effects^76,77^. Often selective pressures are inferred based on correlations between the relative abundances of microbial taxa and geochemical parameters (e.g., toxic stressors or nutrients), although correlative analysis does not identify causal relationships, and observed correlations can be misleading. Thus, our results indicated that fluoride might be critical factor which can shape bacterial communities in groundwater.

## Conclusion

Our study revealed a significant association between fluoride concentration and the composition of bacterial communities in shallow groundwater in the Qiji area, northern China. TOC and fluoride concentration plays an important role in modulating the groundwater bacterial communities. These findings give us new insights into the biogeochemical processes of fluoride and other elements in groundwater, suggesting that fluoride concentration should be considered in future when evaluating microbial response to environmental conditions in groundwater system, especially for fluoride rich groundwater.

## Supplementary information

Supplementary Information

## References

[1] Berbasova T (2017). Fluoride export (FEX) proteins from fungi, plants and animals are ‘single barreled’ channels containing one functional and one vestigial ion pore. PloS one..

[2] Yiamouyiannis JA, Burk D (1976). Fluoridation of public water systems and cancer death rates in humans. Fed. Proc..

[3] Barbier O, Arreola-Mendoza L, Razo LMD (2010). Molecular mechanisms of fluoride toxicity. Chem-Biol. Interact..

[4] Montagnolli RN (2017). The effects of fluoride based fire-fighting foams on soil microbiota activity and plant growth during natural attenuation of perfluorinated compounds. Environ. Toxicol. Phar..

[5] Marquis RE, Clock SA, Mota-Meira M (2003). Fluoride and organic weak acids as modulators of microbial physiology. FEMS Microbiol. Rev..

[6] Qiu YX (2016). Co-operative effect of exogenous dextranase and sodium fluoride on multispecies biofilms. J. Dent. Sci..

[7] Marquis RE (1995). Antimicrobial actions of fluoride for oral bacteria. Can. J. Microbiol..

[8] Barboza-Silva E, Castro AC, Marquis RE (2005). Mechanisms of inhibition by fluoride of urease activities of cell suspensions and biofilms of *staphylococcus epidermidis*, *streptococcus salivarius*, *actinomyces naeslundii* and of dental plaque. Oral Microbiol. Immun..

[9] Andres CJ, Shaeffer JC, Windeler AS (1974). Comparison of antibacterial properties of stannous fluoride and sodium fluoride mouthwashes. J. Dent. Res..

[10] Fine DH, Sreenivasan PK, McKiernan M, Tischio‐Bereski D, Furgang D (2012). Whole mouth antimicrobial effects after oral hygiene: comparison of three dentifrice formulations. J. Clin. Periodontol..

[11] Tinanoff N, Klock B, Camosci DA, Manwell MA (1983). Microbiologic effects of SnF_2_ and NaF mouthrinses in subjects with high caries activity: results after one year. J. dent. Res..

[12] Zorina SY, Pomazkina LV, Lavrent’eva AS, Zasukhina TV (2010). Humus status of different soils affected by pollution with fluorides from aluminum production in the Baikal region. Contemp. Probl. Ecol..

[13] Wilke BM (1987). Fluoride-induced changes in chemical properties and microbial activity of mull, moder and mor soils. Biol. Fert. Soils.

[14] Reddy MP, Kaur M (2008). Sodium fluoride induced growth and metabolic changes in Salicornia brachiata Roxb. Water Air Soil Poll..

[15] Yadu B, Chandrakar V, Keshavkant S (2016). Responses of plants to fluoride: an overview of oxidative stress and defense mechanisms. Fluoride.

[16] Rao DN, Pal D (1978). Effect of fluoride pollution on the organic matter content of soil. Plant Soil.

[17] Cronin SJ, Manoharan V, Hedley MJ, Loganathan P (2000). Fluoride: a review of its fate, bioavailability, and risks of fluorosis in grazed-pasture systems in New Zealand. New Zeal. J. Agr. Res..

[18] Decker EM, Bartha V, Kopunic A, Von OC (2017). Antimicrobial efficiency of mouth rinses versus and in combination with different photodynamic therapies on periodontal pathogens in an experimental study. J. Periodontal Res..

[19] Mendes GDO (2014). Biochar enhances Aspergillus niger rock phosphate solubilization by increasing organic acid production and alleviating fluoride toxicity. Appl. Environ. Microbiol..

[20] Ochoa-Herrera V (2009). Toxicity of fluoride to microorganisms in biological wastewater treatment systems. Water Res..

[21] Pedersen JT, Falhof J, Ekberg K, Buch-Pedersen MJ, Palmgren M (2015). Metal Fluoride Inhibition of a P-type H^+^ Pump: stabilization of the phosphoenzyme intermediate contributes to post-translational pump activation. J. Biolog. Chem..

[22] Sturr MG, Marquis RE (1990). Inhibition of proton-translocating ATPases of *Streptococcus mutans* and *Lactobacillus casei* by fluoride and aluminum. Arch. Microbiol..

[23] Bunick FJ, Kashket S (1981). Enolases from fluoride-sensitive and fluoride-resistant *streptococci*. Infect. Immun..

[24] Song C (2017). Sodium fluoride induces nephrotoxicity via oxidative stress-regulated mitochondrial SIRT3 signaling pathway. Sci. Rep..

[25] Thibodeau EA, Keefe TF (1990). pH-dependent fluoride inhibition of catalase activity. Molecular Oral Microbiol..

[26] Forbes S, Latimer J, Sreenivasan PK, McBain AJ (2016). Simultaneous assessment of acidogenesis-mitigation and specific bacterial growth-inhibition by dentifrices. PloS One.

[27] Guha-Chowdhury N, Iwami Y, Yamada T (1997). Effect of low levels of fluoride on proton excretion and intracellular pH in glycolysing streptococcal cells under strictly anaerobic conditions. Caries Res..

[28] Bowen WH, Hewitt MJ (1974). Effect of fluoride on extracellular polysaccharide production by *streptococcus mutans*. J. Dent. Res..

[29] Ma H (2014). Effects of fluoride on bacterial growth and its gene/protein expression. Chemosphere.

[30] Wegman MR, Eisenberg AD, Curzon MEJ, Handelman SL (1984). Effects of fluoride, lithium, and strontium on intracellular polysaccharide accumulation in *S*. *mutans* and *A*. *viscosus*. J. Dent. Res..

[31] Mendes GDO (2013). Inhibition of Aspergillus niger phosphate solubilization by fluoride released from rock phosphate. Appl. Environ. Microbiol..

[32] Bradshaw DJ, Mckee AS, Marsh PD (1990). Prevention of population shifts in oral microbial communities *in vitro* by low fluoride concentrations. J. Dent. Res..

[33] Daesslé LW (2009). Fluoride, nitrate and water hardness in groundwater supplied to the rural communities of Ensenada County, Baja California, Mexico. Environ. Geol..

[34] Jacks G, Bhattacharya P, Chaudhary V, Singh KP (2005). Controls on the genesis of some high-fluoride groundwaters in India. Appl. Geochem..

[35] Kut KMK, Sarswat A, Srivastava A, Pittman CU, Mohan D (2016). A review of fluoride in african groundwater and local remediation methods. Groundwater Sust. Develop..

[36] Souza CFMD (2013). Assessment of groundwater quality in a region of endemic fluorosis in the northeast of Brazil. Environ. Monit. Assess..

[37] Chapelle F. H. Ground-water microbiology and geochemistry. John Wiley and Sons: New York (1993).

[38] Griebler C, Lueders T (2009). Microbial biodiversity in groundwater ecosystems. Freshwater Biol..

[39] Gao XB, Wang YX, Li YL, Guo QH (2007). Enrichment of fluoride in groundwater under the impact of saline water intrusion at the salt lake area of Yuncheng basin, northern China. Environ. Geol..

[40] Khair AM, Li CC, Hu QH, Gao XB, Wanga YX (2014). Fluoride and arsenic hydrogeochemistry of groundwater at Yuncheng Basin, Northern China. Geochem. Int..

[41] Li CC, Gao XB, Wang YX (2015). Hydrogeochemistry of high-fluoride groundwater at Yuncheng Basin, northern China. Sci. Total Environ..

[42] Wen DG (2013). Arsenic, fluoride and iodine in groundwater of China. J. Geochem. Explor..

[43] WHO. Fluoride in drinking water-background document for development of WHO guidelines for drinking water quality. Geneva: WHO. (2004).

[44] Bowden HW (1990). Effects of fluoride on the microbial ecology of dental plaque. J Dent Res..

[45] Li DN, Gao XB, Wang YX, Luo WT (2018). Diverse mechanisms drive fluoride enrichment in groundwater in two neighboring sites in northern china. Environ. Pollut..

[46] Gao XB, Wang YX, Wu PL, Guo QH (2010). Trace elements and environmental isotopes as tracers of surface water–groundwater interaction: a case study at Xin’an karst water system, Shanxi Province, Northern China. Enviro. Earth Sci..

[47] Gao XB, Zhang FC, Wang C, Wang YX (2013). Coexistence of high fluoride fresh and saline groundwaters in the Yuncheng Basin, northern China. Procedia Earth Planet. Sci..

[48] Luo WT, Gao XB, Zhang X (2018). Geochemical processes controlling the groundwater chemistry and fluoride contamination in the Yuncheng Basin, China—An area with complex hydrogeochemical conditions. PloS one.

[49] Peiffer KH, Sarrazin C (2013). The importance of HCV RNA measurement for tailoring treatment duration. Digest. Liver Dis..

[50] Edgar RC (2013). UPARSE: highly accurate OTU sequences from microbial amplicon reads. Nat. Methods.

[51] Caporaso JG (2010). QIIME allows analysis of high-throughput community sequencing data. Nat. Methods.

[52] Edgar RC, Haas BJ, Clemente JC, Quince C, Knight R (2011). UCHIME improves sensitivity and speed of chimera detection. Bioinformatics.

[53] Haas BJ (2011). Chimeric 16S rRNA sequence formation and detection in Sanger and 454-pyrosequenced PCR amplicons. Genome Res..

[54] Wang Q, Garrity GM, Tiedje JM, Cole JR (2007). Naive Bayesian classifier for rapid assignment of rRNA sequences into the new bacterial taxonomy. Appl. Environ. Microbiol..

[55] DeSantis TZ (2006). Greengenes, a chimera-checked 16S rRNA gene database and workbench compatible with ARB. Appl. Environ. Microb..

[56] Fagervold SK (2014). River organic matter shapes microbial communities in the sediment of the Rhône prodelta. ISME J..

[57] Sun WM (2015). Diversity of the sediment microbial community in the Aha watershed (Southwest China) in response to acid mine drainage pollution gradients. Appl. Environ. Microbiol..

[58] Wang S (2013). Control of temperature on microbial community structure in hot springs of the Tibetan Plateau. PLoS One.

[59] Abercrombie HJ, Skippen GB, Marshall DD (1987). F-OH substitution in natural tremolite, talc, and phlogopite. Contrib. Mineral. Petr..

[60] Zhu C, Sverjensky DA (1991). Partitioning of F-Cl-OH between minerals and hydrothermal fluids. Geochim. Cosmochim. Ac..

[61] Schoeman JJ, MacLeod H (1987). The effect of particle size and interfering ions on fluoride removal by activated alumina. Water SA..

[62] Rafique T (2009). Geochemical factors controlling the occurrence of high fluoride groundwater in the Nagar Parkar area, Sindh, Pakistan. J. Hazard. Mater..

[63] Li P (2017). Analysis of the functional gene structure and metabolic potential of microbial community in high arsenic groundwater. Water Res..

[64] Fierer N, Jackson RB (2006). The diversity and biogeography of soil bacterial communities. P. Natl. Acad. Sci. USA.

[65] Xu M (2010). Responses of microbial community functional structures to pilot-scale uranium *in situ* bioremediation. ISME J..

[66] Baxter NJ (2008). Anionic charge is prioritized over geometry in aluminum and magnesium fluoride transition state analogs of phosphoryl transfer enzymes. J. Am. Chem. Soc..

[67] Pandit S, Kim HJ, Song KY, Jeon JG (2013). Relationship between fluoride concentration and activity against virulence factors and viability of a cariogenic biofilm: *in vitro* study. Caries Res..

[68] Oni OE (2015). Microbial communities and organic matter composition in surface and subsurface sediments of the Helgoland mud area, North Sea. Front. Microbiol..

[69] An YL (2012). Field scale analysis on structural changes of microbial community and its relationships with environmental factors in nitrobenzene-contaminated groundwater during air sparging remediation. Environ. Eng. Manag. J..

[70] Evdokimova GA, Zenkova IV (2003). Effects of aluminum plant emissions on soil biota in the Kola Peninsula. Eurasian Soil Sci..

[71] Baker JL (2012). Widespread genetic switches and toxicity resistance proteins for fluoride. Science..

[72] Banerjee G (2016). Isolation and characterization of fluoride resistant bacterial strains from fluoride endemic areas of west Bengal, India: assessment of their fluoride absorption efficiency. Fluoride..

[73] Praveen KV, Hari PVR (2013). Molecular characterization of fluorine degrading bacteria from soil samples for its industrial exploitation. Int J Adv Life Sci..

[74] Men X (2016). Identification of anion channels responsible for fluoride resistance in oral Streptococci. PloS one.

[75] Carlson HK (2019). The selective pressures on the microbial community in a metal-contaminated aquifer. ISME J.

[76] Tripathi BM (2018). Soil pH mediates the balance between stochastic and deterministic assembly of bacteria. ISME J.

[77] Dini-Andreote F, Stegen JC, Van Elsas JD, Salles JF (2015). Disentangling mechanisms that mediate the balance between stochastic and deterministic processes in microbial succession. Proc. Natl. Acad. Sci. USA.

