# Supplementary material for: Fluoride contributes to the shaping of microbial community in high fluoride groundwater in Qiji County, Yuncheng City, China
Source: Sci Rep. 2019 Oct 9;9:14488. doi: 10.1038/s41598-019-50914-6 (PMC6785547; doi:10.1038/s41598-019-50914-6)
Supplement: Supplementary file 1 — Supplementary Information [file 41598_2019_50914_MOESM1_ESM.docx]

**Supplementary Information**

**Fluoride contributes to the** **shaping of microbial community in high fluoride groundwater in Qiji County, Yuncheng City, China**

Xin Zhang^1^, Xubo Gao^1,*^, Chengcheng Li^1^, Xuesong Luo^2^, Yanxin Wang^1^

1 School of Environmental Studies, China University of Geosciences, Wuhan 430074, P.R. China

2 College of Resources and Environment, Huangzhong Agriculture University, Wuhan, 430070, China

Corresponding authors:

Xubo Gao1,*, Tel: +86-18971476776, E-mail: xubo.gao.cug@gmail.com, Postal address: No. 388, Lumo Road, 430074 Wuhan, China

**Supplementary Table 1.**

Results of Pearson correlation coefficient of the chemical parameters from the groundwater samples.

|  | pH | Depth | EC | HCO_3_^-^ | NO_3_^-^ | Cl^-^ | Ca^2+^ | Mg^2+^ | SO_4_^2-^ | Na^+^ | K^+^ | F^-^ | TOC | TN | TP |
| --- | --- | --- | --- | --- | --- | --- | --- | --- | --- | --- | --- | --- | --- | --- | --- |
| pH |  |  |  |  |  |  |  |  |  |  |  |  |  |  |  |
| Depth | 0.417 |  |  |  |  |  |  |  |  |  |  |  |  |  |  |
| EC | -0.906^**^ | -0.524 |  |  |  |  |  |  |  |  |  |  |  |  |  |
| HCO_3_^-^ | -0.870^**^ | -0.431 | 0.961^**^ |  |  |  |  |  |  |  |  |  |  |  |  |
| NO_3_^-^ | -0.383 | -0.51 | 0.344 | 0.255 |  |  |  |  |  |  |  |  |  |  |  |
| Cl^-^ | -0.907^**^ | -0.545 | 0.995^**^ | 0.934^**^ | 0.339 |  |  |  |  |  |  |  |  |  |  |
| Ca^2+^ | -0.912^**^ | -0.555^*^ | 0.841^**^ | 0.807^**^ | 0.517 | 0.846^**^ |  |  |  |  |  |  |  |  |  |
| Mg^2+^ | -0.916^**^ | -0.539 | 0.989^**^ | 0.967^**^ | 0.417 | 0.977^**^ | 0.861^**^ |  |  |  |  |  |  |  |  |
| SO_4_^2-^ | -0.906^**^ | -0.507 | 0.979^**^ | 0.912^**^ | 0.261 | 0.989^**^ | 0.845^**^ | 0.950^**^ |  |  |  |  |  |  |  |
| Na^+^ | -0.884^**^ | -0.497 | 0.988^**^ | 0.939^**^ | 0.231 | 0.991^**^ | 0.815^**^ | 0.958^**^ | 0.994^**^ |  |  |  |  |  |  |
| K^+^ | -0.693^**^ | -0.404 | 0.788^**^ | 0.808^**^ | 0.565^*^ | 0.759^**^ | 0.641^*^ | 0.843^**^ | 0.674^*^ | 0.707^**^ |  |  |  |  |  |
| F^-^ | 0.678^*^ | 0.276 | -0.475 | -0.47 | -0.438 | -0.482 | -0.720^**^ | -0.48 | -0.475 | -0.444 | -0.326 |  |  |  |  |
| TOC | -0.755^**^ | -0.408 | 0.806^**^ | 0.763^**^ | -0.016 | 0.811^**^ | 0.677^*^ | 0.742^**^ | 0.842^**^ | 0.842^**^ | 0.384 | -0.433 |  |  |  |
| TN | -0.383 | -0.51 | 0.344 | 0.255 | 1.000^**^ | 0.339 | 0.517 | 0.417 | 0.261 | 0.231 | 0.565^*^ | -0.438 | -0.016 |  |  |
| TP | 0.733^**^ | 0.543 | -0.643^*^ | -0.601^*^ | -0.218 | -0.681^*^ | -0.715^**^ | -0.630^*^ | -0.682^*^ | -0.666^*^ | -0.408 | 0.730^**^ | -0.542 | -0.218 |  |
| DO | 0.046 | 0.256 | -0.223 | -0.243 | -0.358 | -0.192 | -0.214 | -0.206 | -0.176 | -0.197 | -0.279 | 0.175 | -0.18 | -0.358 | -0.1 |

Statistical significance is indicated by ** (P<0.01) and * (P<0.05).

**Supplementary Table 2.**

Alpha diversity indices at the 97% OTUs level of groundwater.

| Sample ID | Sequence | Chao1 | Shannon | Coverage |
| --- | --- | --- | --- | --- |
| QJ01 | 45680 | 1889 | 6.65 | 86.93 |
| QJ02 | 22453 | 1836 | 6.46 | 90.19 |
| QJ03 | 16753 | 1810 | 8.59 | 90.70 |
| QJ04 | 25753 | 1594 | 5.76 | 91.31 |
| QJ05 | 13978 | 1666 | 8.37 | 88.75 |
| QJ06 | 17040 | 1569 | 7.16 | 89.65 |
| QJ07 | 35705 | 2699 | 8.17 | 87.05 |
| QJ08 | 18549 | 1807 | 7.72 | 88.29 |
| QJ09 | 25081 | 2184 | 7.67 | 88.70 |
| QJ10 | 30159 | 2052 | 8.09 | 89.63 |
| QJ11 | 20661 | 1977 | 7.76 | 87.95 |
| QJ12 | 35778 | 2208 | 6.41 | 89.58 |
| QJ13 | 22299 | 1851 | 7.81 | 85.42 |

**Supplementary Table 3.**

Results of Pearson correlation coefficient between chemical parameters and the alpha-diversity index.

| Index | pH | Depth | EC | HCO_3_^-^ | NO_3_^-^ | Cl^-^ | Ca^2+^ | Mg^2+^ | SO_4_^2-^ | Na^+^ | K^+^ | F^-^ | TOC | TN | TP | DO |
| --- | --- | --- | --- | --- | --- | --- | --- | --- | --- | --- | --- | --- | --- | --- | --- | --- |
| chao1 | -0.456 | -0.276 | 0.263 | 0.101 | **0.595*** | 0.312 | **0.592*** | 0.255 | 0.323 | 0.245 | 0.093 | **-0.705**** | 0.224 | **0.595*** | -0.42 | -0.093 |
| Shannon | -0.419 | 0.268 | 0.133 | 0.23 | 0.16 | 0.107 | 0.354 | 0.167 | 0.103 | 0.087 | 0.161 | **-0.563*** | 0.235 | 0.16 | -0.236 | 0.01 |

Statistical significance is indicated by ** (P<0.01) and * (P<0.05) and is shown in bold.

**Supplementary Table 4.**

Results of Pearson correlation coefficient between chemical parameters and families that were negatively correlated with fluoride concentration.

|  | pH | Depth | EC | HCO_3_^-^ | NO_3_^-^ | Cl^-^ | Ca^2+^ | Mg^2+^ | SO_4_^2-^ | Na^+^ | K^+^ | F^-^ | TOC | TN | TP | DO |
| --- | --- | --- | --- | --- | --- | --- | --- | --- | --- | --- | --- | --- | --- | --- | --- | --- |
| *Pseudomonadaceae* | -0.149 | -0.191 | -0.044 | -0.11 | 0.528 | -0.027 | 0.301 | -0.028 | -0.039 | -0.089 | -0.124 | **-0.706**** | -0.057 | 0.528 | -0.311 | -0.008 |
| *Caulobacteraceae* | -0.417 | 0.002 | 0.055 | 0.023 | 0.23 | 0.071 | 0.453 | 0.075 | 0.103 | 0.032 | -0.101 | **-0.686**** | 0.253 | 0.23 | -0.331 | 0.22 |
| *Xanthomonadaceae* | -0.226 | 0.19 | -0.044 | 0.074 | 0.034 | -0.074 | 0.237 | -0.026 | -0.069 | -0.08 | -0.097 | **-0.638*** | 0.216 | 0.034 | -0.203 | -0.019 |
| *Methylophilaceae* | **-0.738**** | -0.13 | **0.642*** | **0.713**** | 0.18 | **0.597*** | **0.667*** | **0.663*** | **0.633*** | **0.618*** | 0.376 | **-0.559*** | **0.558*** | 0.18 | -0.422 | -0.087 |
| *Legionellaceae* | **-0.689**** | -0.511 | **0.595*** | **0.613*** | 0.373 | **0.581*** | **0.761**** | **0.631*** | 0.548 | 0.536 | 0.408 | **-0.566*** | **0.572*** | 0.373 | -0.477 | 0.019 |
| *Chthonomonadaceae* | -0.173 | 0.119 | -0.052 | 0.014 | 0 | -0.067 | 0.139 | -0.061 | -0.016 | -0.045 | -0.252 | **-0.603*** | 0.058 | 0 | -0.31 | -0.141 |

Statistical significance is indicated by ** (P<0.01) and * (P<0.05) and is shown in bold.

**Supplementary Table 5.**

Results of Monte Carlo Permutation Test of RDA (permutation=999).

| Factors | pH | TOC | TP | F^-^ | Ca^2+^ |
| --- | --- | --- | --- | --- | --- |
| Correlation coefficient(r^2^) | 0.39 | 0.45 | 0.21 | 0.59 | 0.33 |
| P value | 0.067 | 0.045* | 0.323 | 0.007** | 0.117 |

Statistical significance is indicated by ** (P<0.01) and * (P<0.05) determined by Monte Carlo Permutation Test with 999 permutations.

**
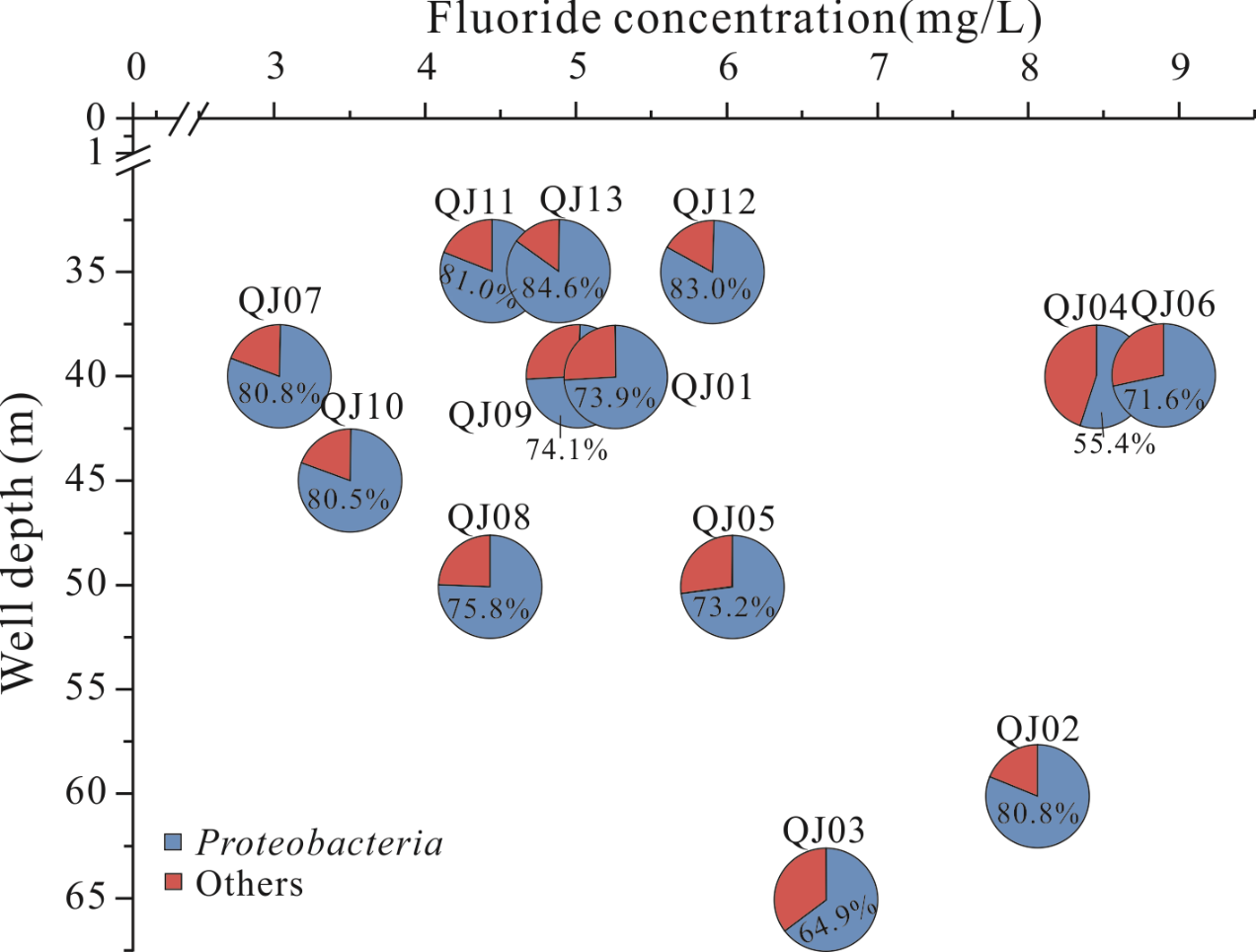
**

**Supplementary Figure 1.** The relative distribution of bacterial populations attached to the fluoride concentration of groundwater samples with different well depths.

**
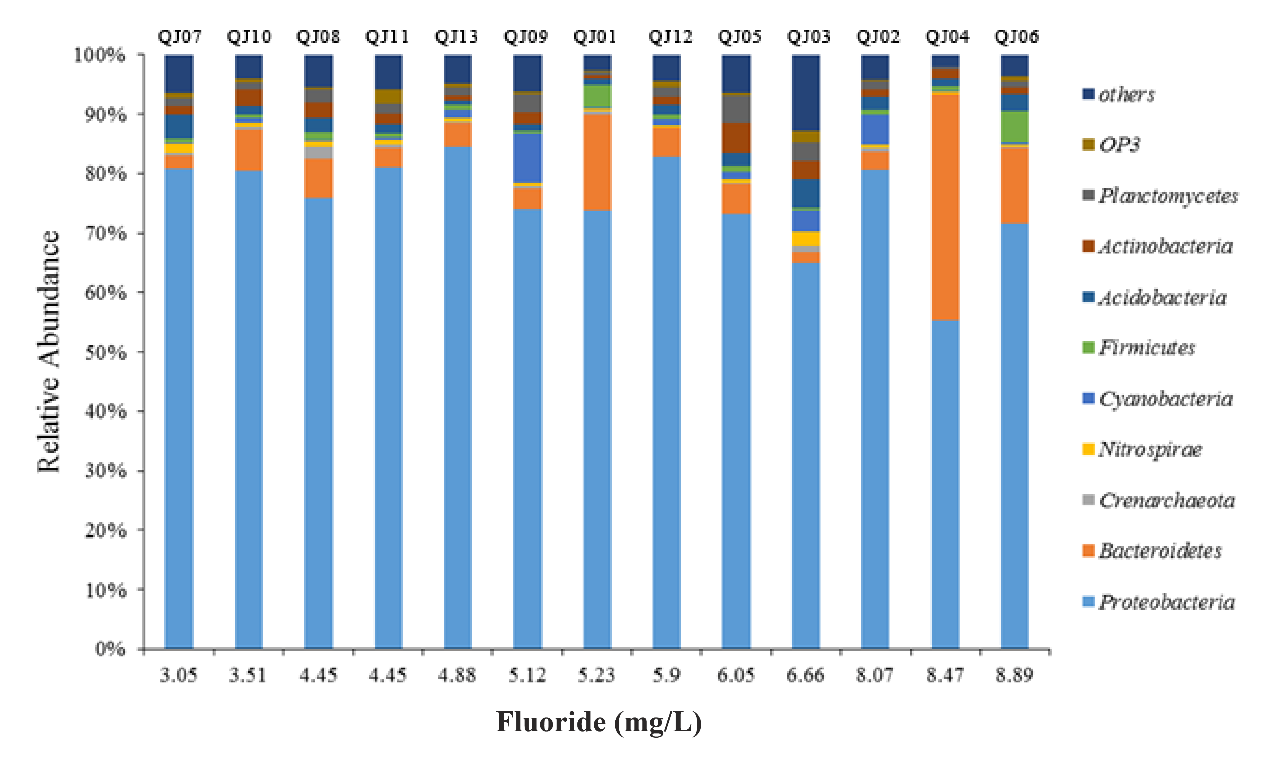
**

**Supplementary Figure 2.** Bacterial community composition of groundwater with varies fluoride concentration at the phylum level. “others” indicates phyla with relative abundances of less than 1.0%.
